# Dysregulation of iron homeostasis and ferroptosis in sevoflurane and isoflurane associated perioperative neurocognitive disorders

**DOI:** 10.1111/cns.14553

**Published:** 2024-02-09

**Authors:** Mengrong Miao, Yaqian Han, Yangyang Wang, Jie Wang, Ruilou Zhu, Yitian Yang, Ningning Fu, Ningning Li, Mingyang Sun, Jiaqiang Zhang

**Affiliations:** ^1^ Department of Anesthesiology and Perioperative medicine People's Hospital of Zhengzhou University, Henan Provincial People's Hospital, People's Hospital of Henan University Zhengzhou Henan Province China

**Keywords:** ferroptosis, iron homeostasis, isoflurane, neurotoxicity, sevoflurane

## Abstract

In recent years, sevoflurane and isoflurane are the most popular anesthetics in general anesthesia for their safe, rapid onset, and well tolerant. Nevertheless, many studies reported their neurotoxicity among pediatric and aged populations. This effect is usually manifested as cognitive impairment such as perioperative neurocognitive disorders. The wide application of sevoflurane and isoflurane during general anesthesia makes their safety a major health concern. Evidence indicates that iron dyshomeostasis and ferroptosis may establish a role in neurotoxicity of sevoflurane and isoflurane. However, the mechanisms of sevoflurane‐ and isoflurane‐induced neuronal injury were not fully understood, which poses a barrier to the treatment of its neurotoxicity. We, therefore, reviewed the current knowledge on mechanisms of iron dyshomeostasis and ferroptosis and aimed to promote a better understanding of their roles in sevoflurane‐ and isoflurane‐induced neurotoxicity.

## Graphical abstract

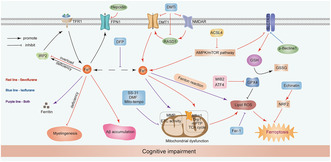

## 1 INTRODUCTION

Perioperative neurocognitive disorders (PNDs) are a group of cognitive disorders regarding to general anesthesia/surgery.^1^ While PND occurred frequently following surgery and anesthesia, determining whether PND results from anesthesia, surgery, or a combination of both remains a challenge to date.^2^, ^3^, ^4^, ^5^ The impact of general anesthesia on neurodevelopment or neurotoxicity was controversial in human studies.^6^, ^7^ But in animal experiments, sevoflurane and isoflurane, two commonly used inhalational anesthetics in general anesthesia, were verified and extensively studied regarding their effect on vulnerable brain such as neonatal or aging brain.^8^, ^9^

Iron, as an essential metal element of every living organism, was involved into many biological processes such as energy metabolism, DNA synthesis, repair, and regulation of signaling pathway.^10^ Disruption of any stage of iron homeostasis including iron uptake, utilization, efflux, and storage may cause abnormal brain function.^11^ Ferroptosis, first proposed by Dixon et al.^12^ in 2012, is a new type of regulated cell death with the characteristics of iron accumulation and lipid peroxidation. In past ten years, ferroptosis was validated with its role in diverse brain disorders involving Alzheimer's disease (AD), Parkinson's disease (PD), stroke, brain aging, brain cancer, and other brain disease.^13^, ^14^

Recently, iron dysregulation and ferroptosis have been implicated in sevoflurane‐ and isoflurane‐induced cognitive impairment. In sevoflurane anesthetized neonatal rats and primary hippocampal neurons, iron overload and ferroptosis were observed in hippocampus and were associated with abnormal cognitive behaviors.^15^ Sevoflurane exposure during pregnancy in mice also led to cognitive impairment in the offspring through causing iron deficiency and inhibiting myelin genesis.^16^ A low‐iron diet significantly alleviated the cognitive deficiency and neurotoxicity induced by sevoflurane anesthesia in old mouse.^17^ In 2019, Xia et al.^18^ found that 6 h of 2% isoflurane exposure caused neuronal cell death, suppressed mitochondrial function, and expression of glutathione peroxidase 4 (GPX4) expression in embryonic mouse primary cortical neuronal cultures, but ferroptosis inhibitor ferrostain‐1 (Fer‐1) reversed these effects. After that, Liu et al.^19^ reported the activation of Beclin1‐mediated ferroptosis in isoflurane anesthetized SH‐SY5Y cells. Also, Fer‐1 rescued isoflurane‐induced cognitive impairment in neonatal mice.^20^

These studies suggested that regulating iron homeostasis and inhibiting ferroptosis in central nervous system (CNS) may offer a potential strategy for mitigating sevoflurane‐ and isoflurane‐induced damage on vulnerable brain during perioperative period. Consequently, this review aims to summarize the current knowledge about iron homeostasis and ferroptosis in the CNS, systematically review their role in sevoflurane‐ and isoflurane‐induced neurotoxicity and offer a new reference for future research.

## 2 IRON METABOLISM IN BRAIN

### 2.1 Iron uptake via BBB

**FIGURE 1:**
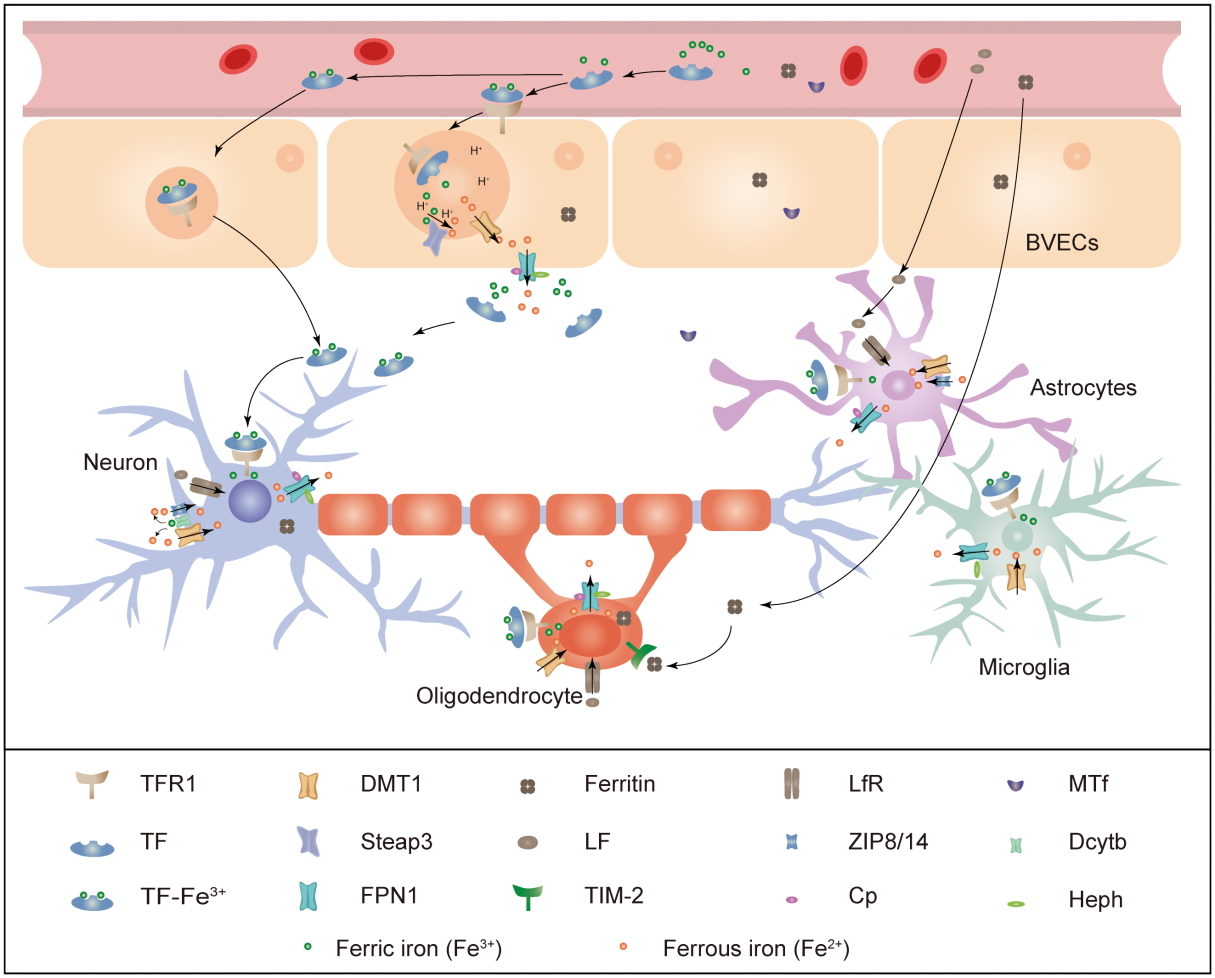
Iron metabolism and regulation in brain. Iron in circulation can cross BVECs of BBB via TF‐TFR pathway, MTf, and LF/LfR pathway. Tf‐TfR complexes are directly transported into the brain at the abluminal membrane of BMVECs or internalized in endosomes. After that, iron can be exported out of BVECs through FPN1. Iron uptake, storage, and exportation within neuron, astrocytes, microglia, and oligodendrocyte are shown in the figure.

Iron performs its biological functions via transferring electrons to participate in oxidation–reduction reactions. Iron in the circulating blood assesses into brain parenchyma by crossing the blood–brain barrier (BBB), it is usually absorbed in the form of transferrin (TF)‐Fe^3+^ and mediated by transferrin receptor (TFR) on the brain vascular endothelia cells (BVECs) of BBB (Figure 1).^21^ Different from transferrin‐bound‐iron (TBI), non‐transferrin‐bound iron (NTBI) emerge in circulation when iron overload and TF was saturated. Fe^2+^ in NTBI is imported into brain cells via divalent metal transporter 1 (DMT1, SLC11A2) or Zinc–Iron‐Proteins 8/14 (ZIP8/14) et al., while Fe^3+^ in NTBI must be transformed to Fe^2+^ by ferrireductases such as six transmembrane epithelial antigen 3 (Steap3) and duodenal cytochrome B (Dcytb) before uptake into cells.^22^ In addition, lactoferrin (LF)/lactoferrin receptor (LfR) pathway also helps to carry iron across the BBB.^23^ As an iron‐binding glycoprotein, LF can exist in cerebrospinal fluid, cross BBB via forming the LfR‐lactoferrin complex with LfR which expressed on the surface of BVECs, and finally transported into the brain interstitium through receptor‐mediated transcytosis.^24^ And Melanotransferrin (MTf) contribute to another Tf‐independent mechanism of brain iron absorption. MTf can bind iron, form a complex with other proteins, and finally enter BVECs through interacting with MTf receptors.^25^

TFR is expressed in the lumen of the brain microvasculature and facilitates the iron transport from circulating blood to BVECs via receptor‐mediated endocytosis.^26^ After endocytosis, Tf‐TfR complexes are directly transported into the brain at the abluminal membrane of BMVECs or internalized in endosomes. And then endosomes are acidificated and release iron to composition of labile iron pool (LIP) or ferritin (FT). NTBI can be absorbed into cells via DMT1 or ZIP8/14.^22^ DMT1 is a transmembrane ion transporter and mediated the transport of various metal ion involving Fe^2+^, Mn^2+^, Co^2+^, and Cd^2+^.^27^ In CNS, DMT1 is widely located in various cells. DMT1 on endothelia cells membrane facilitates the absorption of iron from NTBI to BVECs, while DMT1 located on endosomes is responsible for transporting iron released from TF from endosomes to cytosol with the help of Steap3. In astrocytes, microglia, and neurons, DMT1 also takes part into uptake of Fe^2^ from NTBI.^26^ ZIP8 and ZIP14 are homologous in mammals. ZIP8 is found in cell surface of neurons,^28^ while ZIP14 expresses in astrocytes.^29^

TF/TFR pathway‐mediated iron uptake is the main source of iron transport from BBB and is regulated by iron regulatory proteins (IRPs) at the posttranscriptional level. In the circumstance of iron deficiency, IRPs bind with iron‐responsive elements (IREs) in 3′ untranslated region (UTR) of TFR mRNA to prevent the degradation of TFR mRNA from nucleases. On the contrary, high iron levels inhibit the binding of IRPs and IREs and thus downregulate TFR expression.^30^ In addition, IRPs also suppress the transcription of SLC40A1 and FT through binding to their 5′ UTR.^31^ In 4 alternatively spliced transcripts of DMT1 mRNA, 2 of them found the existence of IREs in 3′ UTR.^32^

When Fe^2+^ is released from endosomes via DMT1, part of it is moved into mitochondria for metabolism by mitoferrin 1 (Mfrn1) and/or 2,5‐dihydroxybenzoic acid (2,5‐DHBA). Intracellular iron is usually stored as ferritin (FT) in the form of ferritin heavy chain (FTH) and ferritin light chain (FTL). When iron in cell is excess, it may be stored in the form of FT in cytosol or transported into labile iron pool (LIP). Iron in BVECs may be ferroportin 1 (FPN1/SLC40A1) is a 66 kDa transmembrane protein which locates at the abluminal membrane of BVECs and mediates the iron transport from BVECs cytosol to brain with the cooperation of ceruloplasmin (Cp) or hephaestin (Heph). Heph and Cp are important multicopper ferroxidases (MCFs) in cellular iron homeostasis of CNS. Heph exists in the epithelial cells of choroid plexus or other brain cells such as neurons, microglia, oligodendrocytes, and astrocytes.^33^, ^34^ CP (ceruloplasmin) is mainly expressed in oligodendrocytes, but also expressed in microglia under certain conditions including brain injury or inflammation.^35^ Cp presents as the form of glycosylphosphatidylinositol‐linked CP (GPI‐CP) in astrocytes.^36^ It facilitates the action of FPN1 via maintaining stability of FPN1 in membrane of cells.^37^ Although Cp is not expressed in neurons, neurons can take up it from the surrounding environment or nearby glial cells.^38^ When Fe^2+^ exported from BVECs by FPN1, it could be converted to Fe^3+^ under the action of Heph or Cp. Studies showed that Heph and Cp play distinct but interrelated roles in regulation of iron metabolism in CNS.^39^ The regulation of FPN1 usually is performed by the binding of hepcidin, which can induce its internalization and degradation in post‐translationally level.^40^

### 2.2 Iron transport within brain

Iron transport within brain can be divided into two forms: TF‐bound iron (TBI) and non‐transferrin‐bound iron (NTBI). After released by BVECs and across the BBB, iron may bind with TF which secreted by the epithelial cells of the choroid plexus. It is reported that TF in CSF and in interstitial fluid (IF) is fully saturated with iron. Iron content in CSF and IF beyond the binding capacity of TF even in normal circumstance, the excess iron exists in the form of NTBI which plays an important role in brain iron balance.^41^ Iron in NTBI can bind with low iron binding affinity compounds such as citrate, ATP, or ascorbate which can secreted by astrocytes.^42^, ^43^ After formation of low molecular weight complexes, NTBI can be taken up by the perivascular end feet of astrocytes.^11^

#### 2.2.1 Iron metabolism in astrocytes

Astrocytes are supposed to be critical in brain iron uptake and homeostasis via interaction with endothelial cells of BBB.^44^ Their end foots surround the BVECs of BBB and also establish a connection to neurons.^45^ Although it is still under debate, the expression of TFR1 in astrocytes was confirmed in recent studies.^44^, ^46^, ^47^, ^48^ Naturally, TFR1 receives the binding of TF and thus bring iron into astrocytes.^49^ Similarly, although controversial, studies confirmed that DMT1 exists in astrocytes.^47^, ^50^ Hence, iron may be absorbed by astrocytes through TF/TFR1 pathway and action of DMT1. Besides, astrocytes may absorb iron in NTBI through mediation of DMT1 or ZIP14.^29^, ^51^ Iron export of astrocytes is mainly performed by FPN1 with the help of Cp.^52^, ^53^

#### 2.2.2 Iron metabolism in neurons

As the main cell type of CNS, neurons are composed of dendrites, axon, and soma. Neurons communicate with each other via synapses and the release of neurotransmitters. Of this process, iron can regulate synaptic plasticity by affecting the activity of N‐methyl‐D‐aspartate (NMDA) receptors or impact synthesis of neurotransmitters involving dopamine, norepinephrine, epinephrine, serotonin, tyrosine as an essential cofactor of their catalyzing enzymes such as tyrosine hydroxylase (TH), tryptophan hydroxylase, and phenylalanine hydroxylase.^54^, ^55^ Equally, the influx of iron in neurons includes multiple pathways. First, TBI forms complex with TFR1 and enter neurons through endocytosis; second, on the surface of neurons membrane, prion protein (PrPC) presents as a ferrireductase to facilitate DMT1‐dependent uptake of Fe^2+^ from NTBI^56^, ^57^, ^58^; third, the involvement of LF/LfR pathway is confirmed in the absorption of iron in neurons.^59^ Iron in cytoplasm of neurons may be utilized by normal need of metabolism or stored in the form of FT. For iron efflux, FPN1 is highly expressed in neurons. Both Heph and Cp express in neurons and help to export iron with FPN1 via oxidation of Fe^2+^ to Fe^3+^.

#### 2.2.3 Iron metabolism in oligodendrocytes

In CNS, iron serves as an important cofactor for enzymes involved in the process of myelin synthesis.^60^ Corresponding to high metabolic need during myelination process, oligodendrocytes was reported with the highest level of iron in brain.^61^ The expression of TFR1 and DMT1 in oligodendrocytes was confirmed in neonatal rats but not in mature brain of adult rats/mice, which indicates that oligodendrocytes may intake iron via TF/TFR1/DMT1 pathway during their developmental process.^62^, ^63^ The main source of iron for oligodendrocytes is FTH, which can enter through interaction with T‐cell immunoglobulin mucin domain 2 (TIM2) receptor.^64^ Iron efflux from oligodendrocytes is mediated by FPN1 and ferroxidase Heph.^34^, ^65^ Interestingly, recent study reported that ferroxidase effect of Cp is also essential for oligodendrocytes maturation and iron homeostasis.^35^

#### 2.2.4 Iron metabolism in microglia

Microglia are mainly deemed as the immune cells in the brain now. Microglia participate the development of many physiological and pathological processes. Microglia can uptake iron through TF/TFR/DMT1 pathway, TF‐independent pathway, and export iron through interaction between FPN1 and Heph^66^ (Figure 1).

## 3 THE KEY MECHANISMS OF FERROPTOSIS IN BRAIN

Ferroptosis, distinct from other classic cell death such as apoptosis, autophagy, and necrosis,^12^ is widely studied in neurological diseases during the past ten years. Cellular iron accumulation and lipid peroxidation of cell membranes are the basic characteristics of cell ferroptosis.^67^ In morphology, cell undergoing swelling, increased mitochondrial membrane density, rupture of the outer membrane, and decreased mitochondrial cristae upon facing the incidence of ferroptosis.^68^

### 3.1 Iron metabolism in ferroptosis

Increasing studies revealed that dysregulation of iron metabolism is associated with ferroptosis. In two forms of iron (Fe^2+^ and Fe^3+^), Fe^2+^ is important for the incidence of ferroptosis. In 1876, Fenton reaction was proposed to clarify the interaction between iron salts and peroxides.^69^ From the formula of Fenton reaction (Fe^2+^ + HOOH/Fe^3+^ + OH^−^ + OH·), yield of toxic hydroxyl radicals could be seen. Correspondingly, iron overload and ferroptosis could be observed in many neurological diseases such as Alzheimer's disease (AD), Parkinson's disease (PD), hemorrhagic, and ischemic stroke.^70^ It has been reported that overexpression of miR‐214 inhibited ferroptosis in brain ischemia/reperfusion (I/R) model via decreasing TFR1 and cellular iron content.^71^ In early brain injury model following subarachnoid hemorrhage, inhibition of hepcidin could regulate iron metabolism and reduce ferroptosis via DMT1 signaling.^72^ I/R injury was alleviated via ferroptosis inhibitory effect of nuclear receptor coactivator 4 (NCOA4) deletion.^73^ In other words, regulation of iron transporters may facilitate to balance iron homeostasis and thus inhibiting ferroptosis in brain.^74^ In addition, iron chelators including deferiprone (DFP) or deferoxamine (DFO) can protect brain from ferroptosis by decreasing iron content and inhibiting the downstream pathways.^75^, ^76^ In summary, regulating iron metabolism‐related proteins or chelating iron accumulation in brain can inhibit Fenton reaction and present protective effect on ferroptosis‐induced brain damage.

### 3.2 Amino acid metabolism in ferroptosis

Amino acid metabolism such as cystine and glutamate is one of the most critical mechanisms for ferroptosis. As transmembrane amino acid transporter, system Xc^−^ intakes cystine and exports glutamate from cell in 1:1 ratio by its subunit SLC7A11 and SLC3A2.^77^ Cystine transported into cell can be reduced to cysteine, cysteine binds with glutamic acid and glycine and thus contributes to the synthesis of glutathione (GSH) which functions its antioxidant role as substrate of glutathione peroxidase 4 (GPX4). As a key regulator of ferroptosis, GPX4 inhibits lipid peroxides by converting GSH to oxidized glutathione (GSSG) and reducing cytotoxic polyunsaturated fatty acids phospholipid hydroperoxides (PUFA‐PL‐OOH) to non‐cytotoxic PUFA phospholipid alcohols (PUFA‐PL‐OH).^78^ Selenium utilization by GPX4 is required to prevent hydroperoxide‐induced ferroptosis.^79^ As a selenoprotein, selenocysteine serves as an important part in redox active role of GPX4.^80^ Inhibiting cystine‐glutamate exchange function of system Xc^−^ triggers ER stress and ferroptosis.^81^ Activating transcription factor 4 (ATF4) knockdown renders cells susceptible for erastin, sorafenib, and RSL3‐induced ferroptosis in system Xc‐ manner.^82^ Ablation of GPX4 induces neuron damage and leads to cognitive impairment and neurodegeneration.^83^ In contrast, activation of the SLC7A11/GPX4 axis presents a protective effect on neurons.^84^ In addition, pretreatment of selenium compounds also exerts anti‐ferroptosis effect.^85^

### 3.3 Lipid metabolism in ferroptosis

**FIGURE 2:**
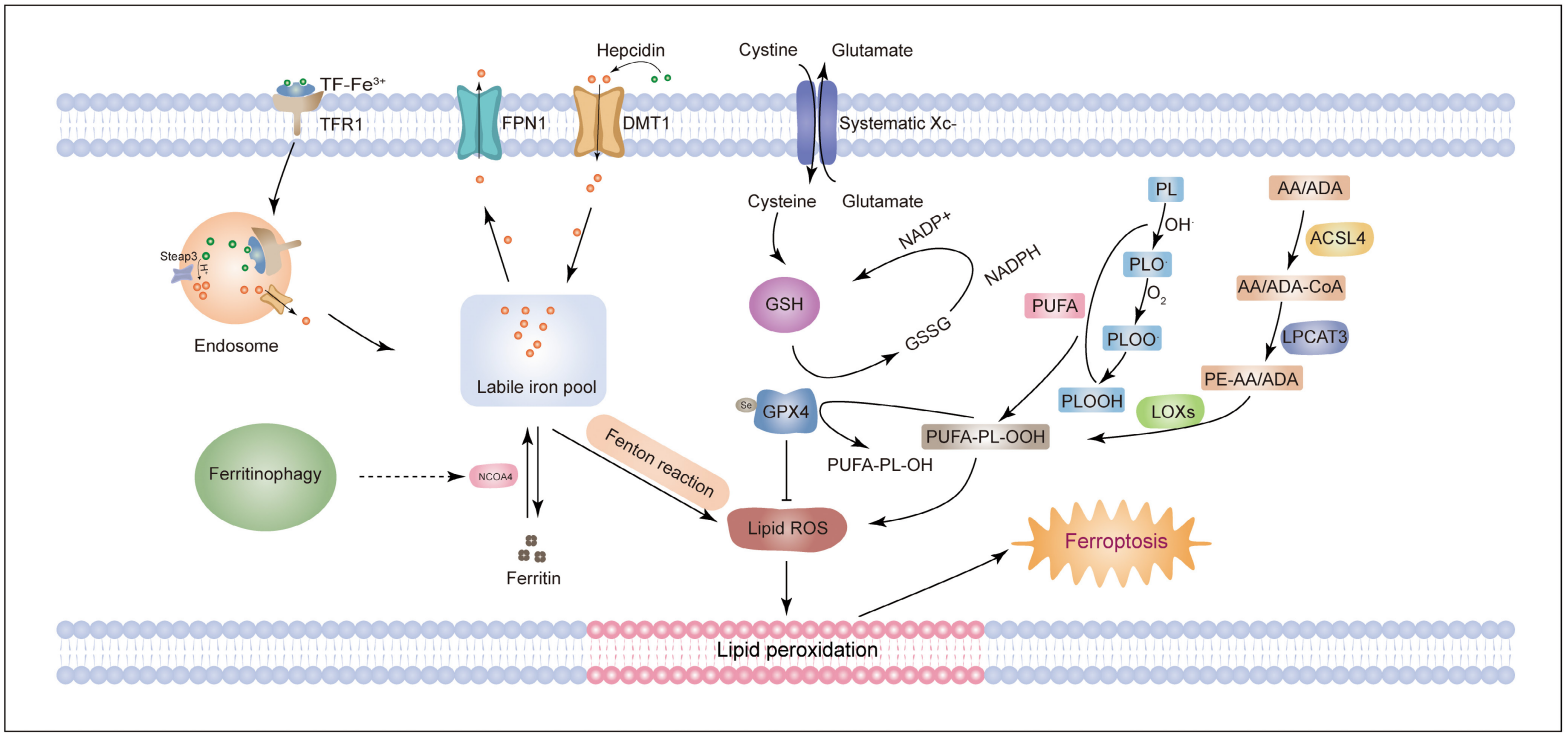
The key mechanisms of ferroptosis in brain. Iron metabolism plays an important role in ferroptosis. Action on various iron transporters involving TFR1, FPN1, and DMT1 may increase intracellular iron content and finally leading to lipid ROS accumulation via fenton reaction; Systematic Xc^−^‐mediated amino acid metabolism contributes to synthesis of GSH and thus facilities the antioxidant effect of GPX4; PUFA may be attacked by lipid radicals and lipid peroxyl radicals generated under this condition. Another pathway of lipid peroxidation enzyme‐activated (including ACSL4, LPCAT3, and LOXs) conversion of AA/AdA to PUFA‐PL‐OOH.

Lipid metabolism is essential in ferroptosis. Lipid ROS accumulation is a hallmark of ferroptosis. In Fenton reaction, Fe^2+^ is oxidized to Fe^3+^ and thus producing hydroxyl radicals (OH·).^69^ Under the action of OH· and O_2_, PUFAs are attacked by generated lipid radicals and lipid peroxyl radicals and thus lipid peroxidation occurs.^86^ Another pathway of lipid peroxidation is related to phosphatidylethanolamines (PEs), which is enzyme activated.^87^ PEs containing arachidonic acid (AA) or adrenic acid (AdA) are key phospholipids that induce ferroptosis. Under the action of acyl‐CoA synthetase long‐chain family member 4 (ACSL4), AA/AdA is converted to AA/AdA‐CoA.^88^ Then AA/AdA‐CoA can be inserted into lysophospholipid PE by lysophosphatidylcholine acyltransferase 3 (LPCAT3) for synthesis of phosphatidylethanolamine‐adrenic acid/arachidonic acid (PE‐AA/AdA).^88^ Finally, PE‐AA/AdA is oxidative to PE‐AA/AdA‐OOH by lipoxygenases (LOXs) and affects the transmembrane characteristics of PUFAs.^87^ GPX4 interrupts this process by reducing PUFA‐PL‐OOH to PUFA‐PL‐OH, and thereby alleviating membrane lipid peroxidation and ferroptosis (Figure 2).

## 4 DYSREGULATION OF IRON HOMEOSTASIS IN SEVOFLURANE‐ AND ISOFLURANE‐INDUCED COGNITIVE IMPAIRMENT

Both sevoflurane and isoflurane presented a neurotoxic role in neonatal and aging brain. Several studies tried to elucidate mechanism of their neurotoxicity from the perspective of iron metabolism.^15^, ^16^, ^17^, ^89^, ^90^ These studies indicated that sevoflurane exposure may disturb the neuronal iron homeostasis, and iron dyshomeostasis in brain can cause cognitive impairment in neonatal and aged rodents. TFR1 is responsible for iron uptake of various neuronal cells. FT is an iron storage protein. As one of critical regulatory proteins in iron metabolism, iron regulatory proteins (IRPs) regulate the expression of TFR1 and FT at the post‐transcriptional level. In neonatal rats and aged mice, multiple sevoflurane inhalation (3% sevoflurane 2 h daily for 3 days) decreased the expression of IRP2 and TFR1 in hippocampus. In this model, sevoflurane anesthesia increased iron level and the expression of FT in hippocampus of neonatal and aged rats.^15^ DMT1 is iron uptake protein for Fe^2+^. NMDAR is an important target of sevoflurane. Through targeting NMDAR, sevoflurane can activate RASD1 and upregulated the expression of DMT1, thus contributing to iron overload in hippocampus of neonatal rats and aged mice. DMT1 inhibitor pretreatment before anesthesia can alleviate sevoflurane‐induced iron overload and cognitive impairment.^15^, ^90^ In accordance with this study, Wang et al. reported the upregulation of FTL, FTH and decrease of TFR1 in hippocampus and cortex of sevoflurane anesthetized aged mice (2% sevoflurane 6 h). Excess iron can promote the aggregation of Aβ via generating ROS, regulating APP (amyloid precursor protein) processing, or directly interacting with Aβ.^91^ And further, iron deficiency treatment alleviated iron‐induced Aβ accumulation.^17^ In sevoflurane anesthetized neonatal mice, Zhang et al.^89^ found the increased level of iron but a decline of FTH in hippocampus. When iron overload occurs after sevoflurane, FPN is upregulated in hippocampus and cortex of aged mice. Hepcidin, a critical protein for internalization and degradation of FPN1, is downregulated.^17^ Iron metabolism dysfunction in brain even could be found in offspring when mice was anesthetized by sevoflurane during pregnancy. In 2020, Zuo et al.^16^ reported sevoflurane anesthesia associated iron deficiency inhibited the growth of oligodendrocyte in and myelinogenesis in hippocampus and cortex of offspring, which could be relieved by iron supplemental therapy.

**FIGURE 3:**
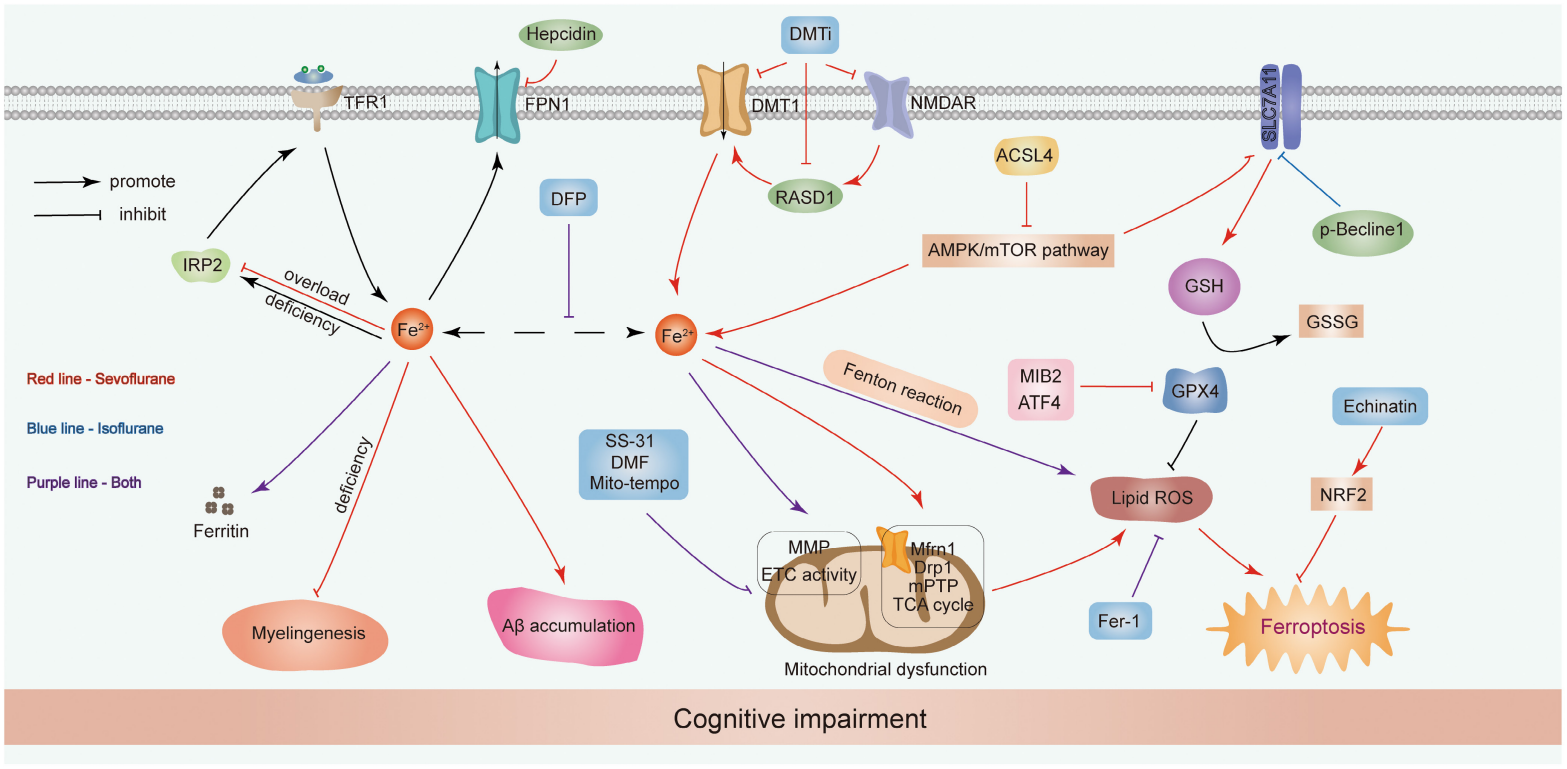
Current knowledge on sevoflurane and isoflurane's effects on iron metabolism and ferroptosis in vulnerable brain. Sevoflurane and isoflurane anesthesia interrupt iron homeostasis and result in ferroptosis in brain. The expression of iron transporters including TFR1, FPN1, and DMT1 is impacted by sevoflurane anesthesia. Sevoflurane anesthesia causes iron overload via NMDAR‐RASD1‐DMT1 pathway‐mediated iron uptake. Iron overload induced by sevoflurane treatment can lead to Aβ accumulation, mitochondrial dysfunction, lipid ROS accumulation, and cognitive impairment in developmental or aging rodents. DFP and Fer‐1 can reverse these negative changes. SLC7A11‐GSH‐GPX4 axis is implicated into sevoflurane and isoflurane induced ferroptosis. Factors regulating this crucial axis are p‐Becline, MIB2, and ATF4. Echinatin inhibits sevoflurane‐induced ferroptosis by upregulatintg NRF2 pathway. In addition, mitochondria protective agents elamipretide (SS‐31), DMF and Mito‐Tempo treatment improved sevoflurane‐ and isoflurane‐induced cognitive deficits.

Although iron‐dependent ferroptosis is observed in isoflurane anesthetized neurons,^18^, ^19^, ^20^ there is little known about the impact of isoflurane on iron transporters and regulation of iron homeostasis in brain to date. In summary, iron homeostasis in brain plays a pivotal role in general anesthesia‐induced cognitive impairment. Iron chelator DFP or low‐iron forage can be effective for treatment of this damage. Similarly, iron deficiency‐related neurotoxicity induced by anesthesia could be prevented by iron supplementation. Targeting iron metabolism‐related proteins such as TFR1, DMT1, hepcidin, and FPN1 may be a therapeutic strategy for anesthesia‐induced brain injury (Figure 3).

## 5 FERROPTOSIS, A NEW TARGET OF SEVOFLURANE AND ISOFLURANE ASSOCIATED PERIOPERATIVE NEUROCOGNITIVE DISORDERS

Ferroptosis is associated with sevoflurane‐ and isoflurane‐induced neurotoxicity in recent years. In hippocampus of neonatal rats and aged mice, elevated levels of iron, ROS, malondialdehyde (MDA), and decreased levels of GSH were observed after sevoflurane exposure. Sevoflurane upregulated mitochondrial fission marker dynamin‐related protein 1 (Drp1) and accelerated mitochondrial fragment and condense in morphology. In function, sevoflurane increased mitochondrial permeability transition pore (mPTP) opening, decreased mitochondrial membrane potential (MMP) level and ATP production, contributing to mitochondrial dysfunction. These ferroptosis‐associated damages involving lipid ROS accumulation, decreased antioxidant capacity, and impaired mitochondrial morphology and function could be relieved by DFP pretreatment.^15^ Sevoflurane treatment increased the Fe^2+^ level, the expression of ASCL4, and decreased the mRNA levels of SLC7A11 and GPX4 in SH‐SY5Y cells. Knockout of ACSL4 inhibited sevoflurane‐induced ferroptosis via the AMPK/mTOR pathway. Hence, ACSL4 was confirmed as a target for sevoflurane‐induced ferroptotic neuronal death.^92^ Mind Bomb‐2 (MIB2), an E3 ubiquitin protein ligase, was found the participation of it in sevoflurane‐induced cognitive impairment through regulating ferroptosis. Sevoflurane anesthesia significantly enhanced the expression of MIB2 in hippocampus of aged mice. An interaction between MIB2 and GPX4 was revealed. Knockdown of MIB2 alleviated the ubiquitination of GPX4 and cognitive injury in aged mice.^93^ The inhibitory effects of sevoflurane on SLC7A11 and GPX4 were also observed by our groups in hippocampal neurons.^94^ Accordingly, sevoflurane aggravated liver transplantation‐induced brain injury in young rats via its ferroptotic effect.^95^ Sevoflurane also caused ferroptosis in glioma cells by activating the transcription factor 4 (ATF4)‐glutathione‐specific gamma‐glutamylcyclotransferases 1 (CHAC1) pathway.^96^

Prenatal sevoflurane exposure presented neurotoxicity in offspring brain development and contributing to cognitive decline in offspring.^16^, ^97^, ^98^ As an important ferroptosis‐regulating transcription factor, the nuclear factor erythroid 2 related factor 2 (Nrf2) also participated in promoting neural stem cell proliferation and differentiation.^99^ Song et al.^100^ found that sevoflurane exposure in third trimester inhibited the proliferation and differentiation of embryonic prefrontal cortex (PFC). In this study, sevoflurane upregulated ROS, TFR1 but downregulated the expression GPX4 and Nrf2 in embryonic PFC. Interestingly, by upregulating the expression of Nrf2, echinatin alleviated sevoflurane anesthesia‐induced oxidative stress and ferroptosis in hippocampal neurons and cognitive deficits in 20‐month‐old rats.^101^ In this study, knockdown of Nrf2 inversed the protective effects of echinatin through regulating iron overload, ROS accumulation, lipid peroxidation, and degradation of GPX4. Nrf2 may also impact the occurrence of ferroptosis by inhibiting anesthesia‐induced neuroinflammatory responses.^102^, ^103^ Hence, Nrf2 may be a potential ferroptosis target for sevoflurane‐induced neurotoxicity in offspring.

The role of ferroptosis in isoflurane neurotoxicity was confirmed by Xia et al.^18^ in 2018. In this study, mouse primary cortical neurons were exposed to 2% isoflurane for 6 h. Isoflurane anesthesia suppressed the expression of GPX4, leading to ROS accumulation, impaired mitochondrial membrane potential, and cell death. Undoubtedly, pretreatment of ferroptosis inhibitor Fer‐1 can mitigate these lesion changes. In SH‐SY5Y neuroblastoma cells, isoflurane induced ferroptosis and affected the activity of SLC7A11 by enhancing Beclin1 phosphorylation and formation of Beclin1‐SLC7A11 complex.^19^ After that, Liu et al.^20^ verified the effect of isoflurane on ferroptosis in vivo. They confirmed the inhibitive effect of isoflurane on system Xc^−^ in hippocampus of neonatal mice.

As an essential organelle for various neurons, ferroptosis‐related morphological changes and dysfunction in mitochondrion can be observed in sevoflurane and isoflurane anesthetized brain.^15^, ^18^, ^20^, ^89^ Sevoflurane anesthesia induced iron overload in mitochondrion, decreased the mitochondrial membrane potential (MMP), triggered the opening of mitochondrial permeability transition pore (mPTP), increased intracellular ROS accumulation, and finally caused mitochondrion dysfunction and ferroptosis. Meanwhile, mitochondria protective agents elamipretide (SS‐31) and Mito‐Tempo treatment improved iron dysmetabolism and ferroptosis through regulating mitochondrion dysfunction and iron accumulation.^15^, ^89^ It was also reported that isoflurane decreased the mitochondrial membrane potential (MMP) and suppressed the activity of Complex IV in mitochondrial electron transport chain (ETC).^18^, ^20^ Pretreatment with Fer‐1 and mitochondria protective agent DMF rescued isoflurane‐induced ferroptosis and learning and memory impairment (Figure 3).

## 6 IRON HOMEOSTASIS, FERROPTOSIS, AND OTHER GENERAL ANESTHETICS

Except for inhalational anesthetics like sevoflurane and isoflurane, other anesthetics mediated general anesthesia also affect iron homeostasis and ferroptosis. General anesthesia may elevate or degrade iron levels or ferroptosis in CNS via targeting iron metabolism‐related proteins or other targets. In 2020, Wu et al.^15^ found that intravenous ketamine caused iron overload of hippocampal neurons both in vivo and in vitro. Ketamine‐induced cognitive impairments in rats were rescued by iron chelator DFP. Conversely, subanesthetic ketamine suppressed chronic restraint stress (CRS) induced ferroptosis by increasing FTH1 and GPX4 levels and decreasingTfr1 levels.^104^ Recently, propofol presented a neuroprotective effect on neuronal ferroptosis. In HT‐22 cells, propofol (50 μM) downregulated erastin‐induced iron accumulation, excess ROS, and lipid peroxidation by upregulating system Xc‐ and GPX4.^105^ Also, due to its potent antioxidant properties, propofol protected against cerebral ischemia reperfusion injury (CIRI) induced ferroptosis via Nrf2/Gpx4 signaling.^106^ In addition, propofol inhibited ischemia/reperfusion (I/R)‐induced ferroptosis in neurons by inhibiting the HIF‐1α/YTHDF1/BECN1 axis.^107^ In contrast to these studies, repeated propofol anesthesia (continuous 200 mg/kg) promoted hippocampal ferroptosis and cognitive dysfunction in aging rats. This effect could be prevented by Avenanthramide‐C through activating Nrf2/ARE pathway activity.^102^ The neurotoxicity of propofol mediated by ferroptosis was also implicated in developing rats. Propofol pretreatment increased Fe^2+^ level, ROS level, MDA content, decreased MMP, and altered expression of ferroptosis‐related proteins of primary hippocampal neurons.^108^ Etomidate treatment before electroconvulsive therapy (ECT) inhibited neuronal ferroptosis in hippocampus through upregulation of BDNF/Nrf2 pathway and thus enhanced the antidepressant effect of ECT.^109^ As a promising neuroprotective anesthetic, dexmedetomidine protected SK‐N‐SH nerve cells from oxidative injury by hindering the elevation of iron and ROS. In this study, dexmedetomidine abolished tert‐butyl hydroperoxide (t‐BHP; an ROS inducer) induced high expression of Tfr1, DMT1, and IRP1/2 by targeting JNK/Sp1 and Stat4/Sp1 signaling.^110^ Dexmedetomidine's ferroptosis inhibiting effect was also confirmed in cerebral ischemia reperfusion injury model in mice, which was abolished by ML385 (a Nrf2 inhibitor).^111^ Liu et al.^112^ found that dexmedetomidine impacted iron metabolism, amino acid metabolism, and lipid peroxidation processes, and thus reduced the damage induced by ferroptosis after intracerebral hemorrhage (ICH). In addition, by activating mTOR‐TFR1 signaling, dexmedetomidine inhibited ferroptosis‐induced damage, thereby enhancing the learning and memory of AD mouse models.

## 7 CONCLUSIONS AND FUTURE PERSPECTIVES

In conclusion, the incidence of iron metabolism and ferroptosis offered a new insight into sevoflurane and isoflurane anesthesia‐induced neurotoxicity during perioperative period. Current knowledge on this topic enhanced clinical significance of anesthesia‐associated ferreous and ferroptotic activities. To date, research on mechanisms of iron metabolism and ferroptosis in inhalational anesthetics‐induced cognitive impairment is still in initial period. This study summarized neurocognitive changes and ferreous and ferroptotic activities associated with sevoflurane and isoflurane anesthesia, these evidence will help to further the investigation and treatment of sevoflurane and isoflurane induced damage on developmental or aging brain in the future.

## AUTHOR CONTRIBUTIONS

Mengrong Miao, Yaqian Han, Mingyang Sun, and Jiaqiang Zhang contributed to the study conception and design. Yangyang Wang, Jie Wang, Ruilou Zhu, Yitian Yang, Ningning Fu, and Ningning Li researched data for the article and contributed to discussion of the content. All authors contributed to the writing of this article and commented on versions of the manuscript.

## FUNDING INFORMATION

This work was supported by the Natural Science Foundation of China (Nos. 82301448, 82071217, 82001147) and Natural Science Foundation of Henan Province (No. 202300410353).

## CONFLICT OF INTEREST STATEMENT

The authors declare no conflict of interest.

## CONSENT FOR PUBLICATION

All the authors have read the manuscript and agreed to give their consent for the publication in cellular and molecular life science.

## Data Availability

All data relevant to this study are included in the text, references, and figures.
